# A novel avian intestinal epithelial cell line: its characterization and exploration as an in vitro infection culture model for *Eimeria* species

**DOI:** 10.1186/s13071-023-06090-8

**Published:** 2024-01-19

**Authors:** Huifang Chen, Juan Li, Xiaoting Pan, Zhichao Hu, Jianfeng Cai, Zijie Xia, Nanshan Qi, Shenquan Liao, Zachary Spritzer, Yinshan Bai, Mingfei Sun

**Affiliations:** 1 Guangdong Provincial Key Laboratory of Animal Molecular Design and Precise Breeding, School of Life Science and Engineering, Foshan University, Foshan, 528225 China; 2 Key Laboratory of Livestock Disease Prevention of Guangdong Province, Key Laboratory of Avian Influenza and Other Major Poultry Diseases Prevention and Control, Ministry of Agriculture and Rural Affairs, Institute of Animal Health, Guangdong Academy of Agricultural Sciences, Guangzhou, 510640 China; 3 Department of Genetics, Perelman School of Medicine, University of Pennsylvania, Philadelphia, PA USA

**Keywords:** Avian embryo, Avian intestinal epithelial cell line (AIEC), *E. tenella*, Culture model

## Abstract

**Background:**

The gastrointestinal epithelium plays an important role in directing recognition by the immune system, and epithelial cells provide the host's front line of defense against microorganisms. However, it is difficult to cultivate avian intestinal epithelial cells in vitro for lengthy periods, and the lack of available cell lines limits the research on avian intestinal diseases and nutritional regulation. Chicken coccidiosis is a serious intestinal disease that causes significant economic losses in the poultry industry. In vitro, some cell line models are beneficial for the development of *Eimeria* species; however, only partial reproduction can be achieved. Therefore, we sought to develop a new model with both the natural host and epithelial cell phenotypes.

**Methods:**

In this study, we use the *SV40* large T antigen (*SV40T*) gene to generate an immortalized cell line. Single-cell screening technology was used to sort positive cell clusters with epithelial characteristics for passage. Polymerase chain reaction (PCR) identification, immunofluorescence detection, and bulk RNA sequencing analysis and validation were used to check the expression of epithelial cell markers and characterize the avian intestinal epithelial cell line (AIEC). AIECs were infected with sporozoites, and their ability to support the in vitro endogenous development of *Eimeria tenella* was assessed.

**Results:**

This novel AIEC consistently expressed intestinal epithelial markers. Transcriptome assays revealed the upregulation of genes associated with proliferation and downregulation of genes associated with apoptosis. We sought to compare *E. tenella* infection between an existing fibroblast cell line (DF-1) and several passages of AIEC and found that the invasion efficiency was significantly increased relative to that of chicken fibroblast cell lines.

**Conclusions:**

An AIEC will serve as a better in vitro research model, especially in the study of *Eimeria* species development and the mechanisms of parasite–host interactions. Using AIEC helps us understand the involvement of intestinal epithelial cells in the digestive tract and the immune defense of the chickens, which will contribute to the epithelial innate defense against microbial infection in the gastrointestinal tract.

**Graphical Abstract:**

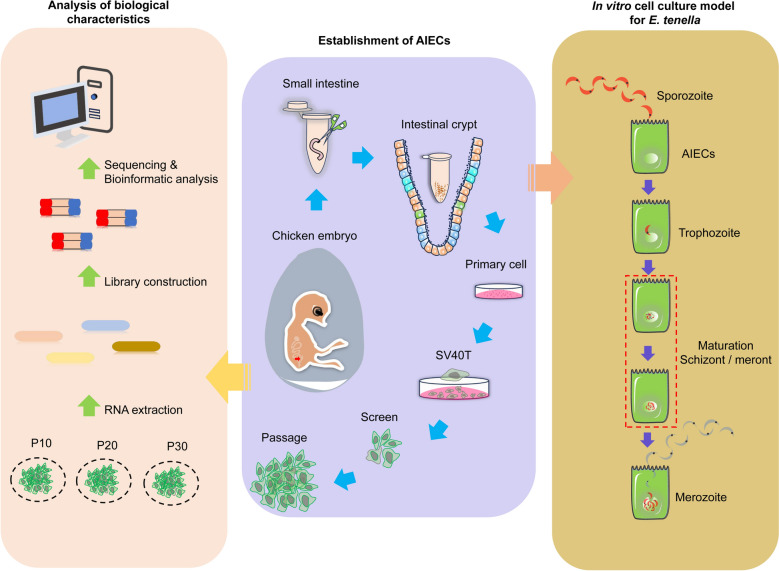

## Background

In the intestine, single-layer epithelial cells serve as the first line of defense, forming structures such as the villi and crypts of Lieberkühn. In addition to its role in nutrient absorption and metabolism, the intestinal epithelium also establishes a mucosal barrier against pathogens [1]. In vitro systems involving cultured intestinal epithelial cells are indispensable for studying the interaction and inflammatory response of intestinal microorganisms [2]. Coccidiosis is a serious intestinal disease caused by protozoans of the genus *Eimeria*, which develop within intestinal epithelial cells and cause varying degrees of morbidity and mortality [3].

This disease results in serious economic damage to the poultry industry, with estimated annual losses amounting to more than $3 billion [3, 4]. The exploration of new strategies is urgently needed to control coccidiosis without inducing drug resistance. However, there is a lack of an effective in vitro culture model, in particular a chicken intestinal epithelial cell model, which can maintain the epithelial cell phenotype and exhibit natural characteristics [5]. This greatly limits research on the mechanisms of pathogenicity and host–pathogen interactions of *Eimeria* spp.

Avian embryo, primary cell, and passage cell models have been used in *Eimeria* culture systems [6]. Long et al. successfully studied avian coccidian infection in an avian embryo culture model for the first time [7]. However, although the chicken embryo culture model exhibits good culture characteristics, it is costly and inconvenient for high-throughput operations. Subsequently, a primary cell culture model was developed. Chicken primary kidney cells and primary cecal epithelial cells were proven to be effective in supporting the development of *Eimeria tenella* sporozoites into oocysts [8, 9], and the biological course of development for *E. tenella* is roughly the same in the primary cell culture model as that in vivo [10]. Nevertheless, primary cells possess a short lifespan due to replicative senescence, making their long-term culture impossible and thus restricting their application. Conversely, cell lines can maintain several characteristics following numerous passages, which not only provides a consistent supply of target cells but also increases the reproducibility of experimental results [11]. Normally, the main method for establishing a cell line is immortalization via the transfection of the *SV40* large T antigen (*SV40T*) gene or the human telomerase reverse transcriptase (*hTERT*) gene [12, 13]. *SV40T* can modify the regulation of the host cell cycle, and *hTERT* initiates telomerase activation and extends the cell lifespan [14]. In comparison, *SV40T* has higher transfection and expression efficiency.

A variety of cell lines have been successfully established from models of porcine, bovine, goat, and other species’ intestinal tissues [5]. For example, duck intestinal epithelial cells were successfully established by transferring the *Lentivirus*-mediated *SV40T* gene into small intestinal epithelial cells derived from duck embryos [15]. Desmarets et al. established a cat intestinal epithelial cell line by transducing *SV40T* and *hTERT* [16]. However, it is more difficult to construct immortalized cell lines from Aves than from mammals because avian species have low natural mutation rates [17]. Commercial poultry-derived cell lines are relatively scarce, and attempts to propagate *E. tenella* in cell line cultures have had limited success. Different cell lines, including bovine kidney cells (MDBK), chicken fibroblast cells (DF-1), human epithelial cells (Caco-2), chicken hepatoma cells (LMH), chicken macrophage-like cells (HD11), chicken lung epithelial cells (CLEC-213), and baby hamster kidney cells (BHK), have been shown to support *E. tenella* infection in vitro [18–21]. Among these, MDBK and DF-1 are widely used for drug screening and parasite–host interactions [22, 23]. Until now, existing cell lines have been unable to support the whole life cycle of *E. tenella*, and instead only supported some asexual stages. Because none of these cell lines comes from natural hosts and target organs, this greatly limits the further development of related research, including the sexual stages of *E. tenella*.

There is currently still no chicken intestinal epithelial cell line in which *Eimeria* can develop to the same stages as within a host. Therefore, a stable and reliable avian intestinal epithelial cell line (AIEC) is needed to facilitate research on coccidian invasion mechanisms, the action of anti-coccidian agents, host–pathogen interaction, and drug screening. In the present study, AIECs were successfully established for the first time, and their epithelial cell phenotype was maintained. We chose *SV40T* to construct and screen the transformed cells using green fluorescent protein (GFP) markers for subculturing. The biological characteristics of AIECs were analyzed using bulk RNA sequencing (RNA-Seq), and their responses to *E. tenella* infection were tested. The expression of most cell cycle-related genes in AIECs was significantly altered in vitro. Our data suggest that AIECs can support the development of the asexual stages of *E. tenella*, and second-generation schizonts were observed. AIECs exhibited a better response to *E. tenella* invasion than DF-1 cells, especially in terms of increasing the invasion rate. This will become a powerful additional research tool for exploring the developmental potential of Coccidia in vitro.

## Methods

### Experimental animals, cells, and parasites

Lingnan Yellow schicken embryos at embryonic (E) ages of E9, E11, E13, E15, and E18 were purchased from Nanhai Breeding Poultry Co., Ltd., Foshan City, Guangdong Province. A total of 750 chicken embryos were used, and five biological replicates were performed each time, with 25 chicken embryos in each group, for six replicates in all.

DF-1 cells (ATCC CRL-12203) were cultured in Dulbecco’s modified Eagle’s medium (DMEM) (Jennio Biotech Co., Ltd, Guangzhou, China) supplemented with 10% fetal bovine serum (FBS, Gibco, USA) and 1% penicillin/streptomycin (PS, Gibco) at 37 °C in a 5% CO_2_ incubator.

The Guangdong (GD) strain of *E. tenella* was isolated and preserved by the Laboratory of Parasitic Biology, Institute of Animal Health, Guangdong Academy of Agricultural Sciences, and propagated every 4 months. Sporulated oocysts of the *E. tenella* GD strain used in this experiment were freshly prepared, and sporozoites were collected and purified from cleaned sporulated oocysts using standard procedures [24, 25]. Freshly purified sporozoites were then used to infect cell monolayers.

All the animal experiments were performed in strict accordance with the recommendations of the Ethical Review Committee (No. PT-2021012) from the Institute of Animal Health, Guangdong Academy of Agricultural Sciences, China.

### Isolation, observation, and culture of primary avian intestinal epithelial cells

Before the experiment, E9, E11, E13, E15, and E18 embryos were pre-cooled for 30 min, surface-disinfected, and transferred to a sterile workbench. Among this group, six embryos were taken from E9 and E11, and three embryos were obtained from E13, E15, and E18. After the eggshell was opened from the air chamber surface, the embryos were removed, washed three times in cold phosphate buffer (PBS, #10010023; Gibco), and then dissected. We separated and obtained 0.5 cm U-segments of the small intestine (duodenum), which were transferred to a 90 mm culture dish (#430167; Corning, USA). The lumen was washed 2–3 times with 10 ml cold 1× PBS, transferred to an Eppendorf (EP) tube, and cut into small fragments with a diameter of 1 mm. Cold 1× PBS was used to wash these tissues with centrifugation of 1000 rpm/min for 3 min, and then the supernatants were discarded. Next, tissues were rinsed using DMEM basal medium (#11960, Gibco) and centrifuged at 1000 rpm/min for 3 min, and the supernatants were discarded. Cell culture was performed by tissue block culture; 15% FBS, 1% GlutaMAX (#35050061, 100×, Gibco), 1% PS (#15140122, 10,000 U/ml, Gibco), 1% insulin transferrin selenium (ITS, #41400045, 100×, Gibco), and basic fibroblast growth factor (bFGF, #10018B, 10 ng/ml, PeproTech/Thermo Fisher Scientific, USA) were added to DMEM basal medium to form DMEM complete medium. Tissue pellets were resuspended in DMEM complete culture medium and transferred to a T25 cell culture flask, with the medium covering the tissue for culture at 37 °C in a 5% CO_2_ incubator. The growth status of the cells was observed at different time points. When the growing cells reached approximately 80% confluence, they were digested with 0.25% trypsin–ethylenediaminetetraacetic acid (EDTA) (#2520007, 1×, Gibco) for 30 s, digestion was terminated, and the cells were collected by centrifugation.

### Immortalization of primary avian intestinal epithelial cells using lentiviral vectors

Virus production was performed following previously published methods [26]. The lentiviral gene transfer plasmids of 10 µg pcDNA-Large T–IRES-Co, along with the package plasmids of 10.4 µg psPAX2 and 3.5 µg pMD2.G, were transfected with 70% confluent 10 cm plates of 293FT embryonic kidney epithelial cells (EY-X0869, ATCC, USA) by PolyFect Transfection Reagent (#301105, Qiagen, Germany). The recombinant lentiviral particles found in the supernatant were collected at 48 and 72 h, followed by 0.45 µm filtration into sterilized 50 ml centrifuge tubes. The high-concentration *Lentivirus* was harvested through an ultra-filtration device (UFC9011096 MilliporeAmicon, Millipore/Merck, Germany) by centrifugation at 4000 rpm for 30 min at 4 °C, and *Lentivirus* aliquots were stored at −80 °C until further use. Viral particles were then suspended in 500 µl AIEC complete medium with 6 µg/ml polybrene (H9268, Sigma, USA), and lentiviral solution at a multiplicity of infection (MOI) of 20 was applied to infect 1 × 10^5^ AIECs after first passage for 6 h. Culturing of infected AIECs was continued for another 48 h. The infection results were analyzed by observing the expression of GFP under a fluorescence microscope (IX73, Olympus, Japan). A clonal population with morphology similar to that of intestinal epithelial cells and high GFP expression was selected using single-cell screening technology. The ability of immortalized cells to survive senescence was confirmed by continuous culture for 20 passages [27]. The established cells were confirmed by continuous culture for over 35 passages and were subsequently referred to as AIECs.

### Total RNA extraction, library construction, RNA-Seq, and quantitative polymerase chain reaction

AIEC passages 10, 20, and 30 (F10, F20, and F30, respectively) were selected for (RNA-Seq), with three biological replicates, respectively. Total RNA was extracted from F10, F20, and F30 using a cell and tissue total RNA extraction kit (#DP451, Tiangen, China) and a reverse transcription first-strand complementary DNA (cDNA) synthesis kit (#K1622, Thermo Scientific, USA). Spectrophotometry (NanoDrop 2000, Thermo Fisher, Waltham, MA, USA) was used to measure the concentration and integrity of the total extracted total RNA, and a library was constructed. An Illumina HiSeq™ 2000 instrument (Illumina, San Diego, CA, USA) was used for sequencing by Biomarker Technologies Co., Ltd.

One microgram of RNA was used as a standard for synthesis of cDNA and was subjected to 1.25% agarose gel electrophoresis. The following polymerase chain reaction (PCR) procedure was used: initial denaturation at 95 °C for 5 min, denaturation at 95 °C for 30 s, annealing at 60 °C for 30 s, extension at 72 °C for 45 s, and final extension at 72 °C for 7 min. The PCR products were stored at 4 °C. Real-time quantitative PCR (qPCR) was performed in a CFX96 Touch^®^ Real-Time PCR Detection System (Bio-Rad, Hercules, CA, USA). TB Green^®^ Premix Ex Taq™ II kit (Tli RNaseH Plus; #RR820A, Takara Bio, Shiga, Japan) was used for the qPCR. The following qPCR procedure was used: 95 °C initial denaturation for 1 min, 95 °C denaturation for 15 s, 60 °C annealing for 15 s, 40 cycles; 95 °C for 10 s; and melting curve at 65–95 °C, increasing 0.5 °C every 5 s.

To evaluate the endogenous development, gene transcription was quantified using cDNA (real-time quantity PCR, qPCR) by measuring the parasite glyceraldehyde 3-phosphate dehydrogenase (GAPDH). Specific primers for the *E. tenella GAPDH* (*EtGAPDH*) and *Gallus gallus GAPDH* (*GallusGAPDH*) genes were used to assess the messenger RNA (mRNA) expression of *EtGAPDH* (target gene) and *GallusGAPDH* (internal reference gene), respectively. The relative expression levels of *EtGAPDH* in the different developmental stages were calculated by the 2^−ΔΔCT^ method [22]. Data were analyzed using Bio-Rad CFX Manager software (Bio-Rad).

All primers used in this study were designed by Primer-BLAST (https://www.ncbi.nlm.nih.gov/), and synthesized by Guangzhou Aiji Biotechnology Co., Ltd. The sequences of all the primers described above are listed in Table 1.

**Table 1:** Summary of target gene primers used for PCR and qPCR analysis

Gene	Primer sequence (5′ to 3′)	Product size (base pairs)	Accession number
*CCNE2*	F: ACCAGGAAAAGAAGAACGGCA	129	NM_001030945.2
	R: GATGATGCAAGGCGAGATGC		
*CDC25A*	F: CTCGCCGGTCTCAGATCTTC	247	XM_001199572.1
	R: TCTTCTGAGAGGCAGTCGGA		
*CDH1*	F: AGCCAAGGGCCTGGATTATG	230	NM_001039258.3
	R: GGTGAATGTACAGCCAGCCT		
*CDK1*	F: CGAGCCTTTGGAATCCCAGT	249	XM_015288063.3
	R: GGATTCCACATCAGGCCACA		
*CDKN1A*	F: AGCAGCTGGAAGAAGCTCAG	289	NM_204396.2
	R: TCTTTGATGGTGGTCTGCCC		
*CLDN1*	F: ACCCACAGCCTAAGTGCTTC	200	NM_001013611.2
	R: AGGTCTCATAAGGCCCCACT		
*GTSE1*	F: GAGGGAGACGTTCTGTGTGG	182	NM_001031332.2
	R: GTTCCTGTGTCAGAGGCGAA		
*KRT-18*	F: GCCAAGAAGAACGTGGAGGA	290	XM_025145666.1
	R: GCGCAGAGCATCCAGGTC		
*OCLN*	F: GTGTAAGGCCCACACCTCTG	187	NM_205128
	R: ATGCCTTCCCAAAAAGCCCT		
*TJP1*	F: ACTGTGACCCCAAAACCTGG	294	XM_040680632.1
	R: CTCCCTGCTTGTGGCATGTA		
*TP53I3*	F: CTTGAGAACTGATGGCCGGT	217	XM_040698015.1
	R: AAATGGCTGGAGATGTGGGG		
*VILL*	F: CGTGCACGTTACCTAACACAG	181	XM_418521
	R: TTACCCACACCCAGTCATGC		
*GallusGAPDH*	F: GGTGGCCATCAATGATCCCT	105	NM_204305.2
	R: CCGTTCTCAGCCTTGACAGT		
*EtGAPDH*	F: TGGAGTCTTCACGAACAAGGA	109	XM_013378585.1
	R: ACCCATCACAAACATCGGAGTA		

### Enrichment analysis of GO and KEGG pathways

Low-quality sequences were filtered out (N > 10%, base number with mass value ≤ 10) from the original data. The DESeq2 package [28] was used to analyze the differentially expressed genes (DEGs) between sample groups, with log_2_ fold change (fold change [FC]) < 1 and adjusted *P*-value < 0.05 as the threshold. Gene Ontology (GO; http://www.geneontology.org/) and Kyoto Encyclopedia of Genes and Genomes (KEGG; http://www.Keg.jp/kegg) significance enrichment analysis was used to describe the biological processes, cell components, and molecular functions to identify the main regulatory genes and signaling pathways.

### Histochemistry

Small intestinal sections were fixed overnight in 4% paraformaldehyde (#P1110, Solarbio, USA), washed 2–3 times with cold PBS, dehydrated in 70% ethanol, and embedded in paraffin. The paraffin blocks were cut into 5 mm sections, which were then deparaffinized and stained with hematoxylin and eosin (H&E). Sections of the small intestine (E9, E11, and E15) were washed with PBS containing 0.1% Tween 20 (#P1379, Sigma) and blocked with 1% bovine serum albumin (BSA, #A104912, Aladdin, China) in PBS containing Tween 20 (PBST) for 1 h. After blocking, sections were incubated with the leucine-rich repeat-containing G-protein-coupled receptor 5 (LGR5)/GPR49 cell marker rabbit polyclonal antibody (1:200, #AF0165, Beyotime Biotechnology, China) overnight at 4 °C. The sections were then washed with PBS and incubated with goat anti-mouse immunoglobulin G (IgG) antibody (#ab150113, 1:2000, Abcam, UK) at room temperature for 1 h. After washing three times in PBS, the stained slides were incubated with 4′,6-diamidino-2-phenylindole (DAPI, #32670, Sigma) diluted 1:500 in PBS for 5 min and imaged.

### Immunofluorescence

Cells were seeded into six-well plates (#3516, Corning, NY, USA) at a density of 2 × 10^5^/ml, and fixed with 4% paraformaldehyde for 10 min at room temperature. The cells were then washed three times with PBS, permeabilized with 0.5% Triton X-100 (#93443, Sigma) for 10 min, and blocked with 1% BSA for 30 min. Cells were immunolabeled with zonula occludens-1 (ZO1; *TJP1*) rabbit polyclonal antibody (#AF0321, 1:500, Beyotime Biotechnology) overnight at 4 °C. The cells were washed with PBS and incubated with goat anti-mouse IgG antibody (Abcam, 1:200) at room temperature for 1 h. After washing three times in PBS, the cells were incubated for 5–10 min with Hoechst 33342 solution (#14533, Sigma) and imaged.

### In vitro parasite development assays

AIEC or DF-1 cells (1.0 × 10^5^ cells) were plated onto cell slides or 24-well plates and cultured at 37 °C in a 5% CO_2_ incubator to cell coverage of 80–90%. Freshly isolated sporozoites were incubated with DMEM (2% FBS, 5% PBS) for 2 h at 37 °C. AIEC or DF-1 cells were infected with pretreated sporozoites with an MOI of 2. At different time points during infection (4, 6, 12, 24, 48, 72, and 96 h), the medium and noninvasive sporozoites were removed by gentle washing three times with PBS. AIEC or DF-1 cells without sporozoite infection were used as the uninfected controls. Cell samples on slides were prepared for H&E staining (for specific steps, refer to the H&E staining kit instructions, #C0105S, Beyotime Biotechnology) to observe the morphology and structure at different stages of development. Every cell sample ready for qPCR was harvested with a cell scraper and stored at −80 °C for subsequent analysis.

### Statistical analysis

All values are given as mean ± standard error (SE). Means of groups were from at least three independent experiments and compared with Student’s t test (unpaired) or the analysis of variance (ANOVA) test when appropriate, and *p* < 0.05 was considered statistically significant (SPSS 17.0, Chicago, IL, USA). Histograms were prepared using the GraphPad Prism software (version 8.0; San Diego, CA, USA). Fluorescence intensity was analyzed using ImageJ software (National Institutes of Health, Bethesda, MD, USA). All results were considered statistically significant at DESeq2 adjusted *P*-value < 0.05 and log_2_(FC) < 1.

## Results

### Morphological assessment of chicken intestine for the establishment of AIECs

**Fig. 1:**
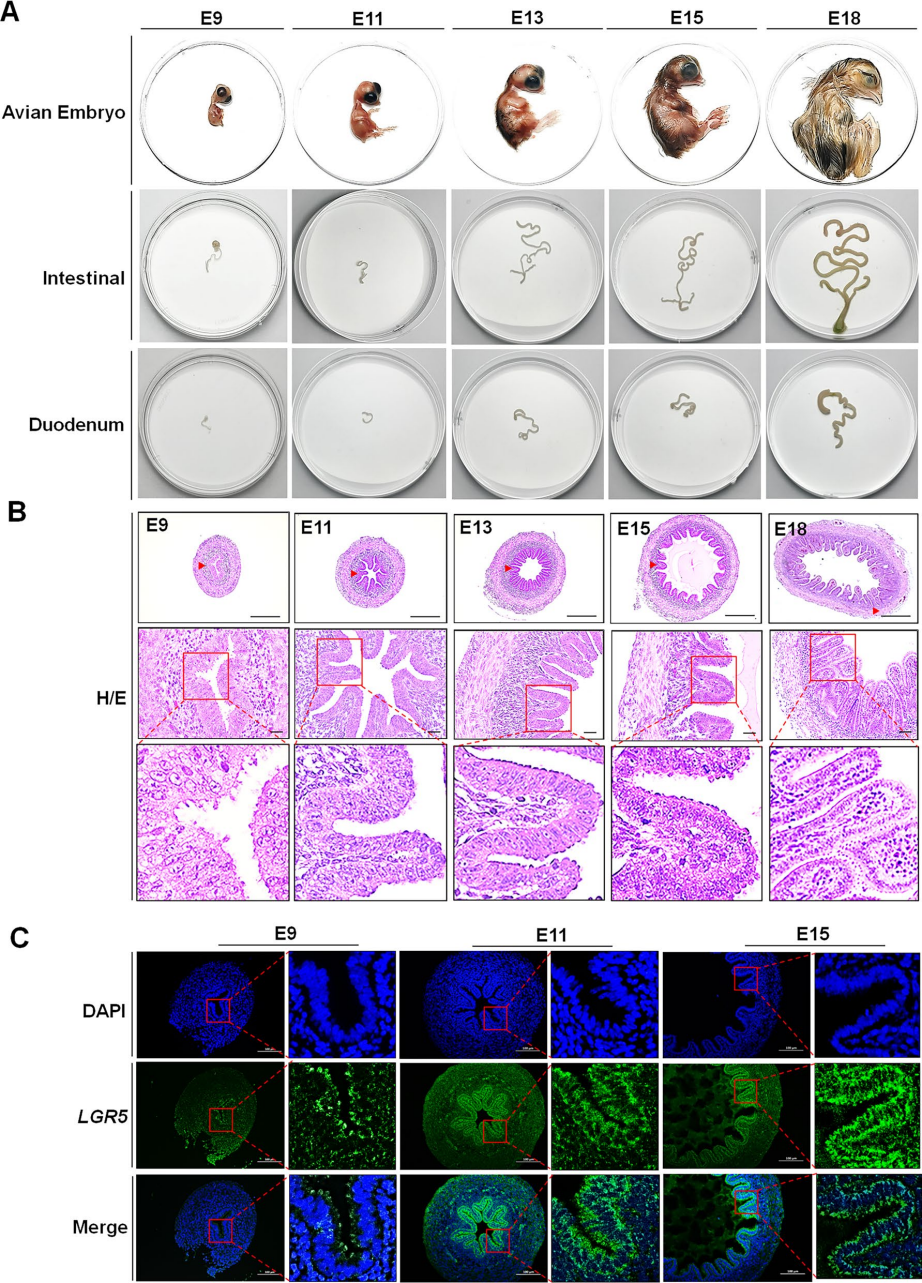
Development of avian embryonic intestinal tissue visualized by hematoxylin and eosin (H&E) histochemistry and immunostaining for marker *LGR5*. **A** Avian embryos of several ages were obtained, and intestinal tissues were isolated to observe their morphology. **B** The development of the small intestine at E9, E11, E13, E15, and E18 were observed by H&E staining. **C** *LGR5* expression by immunofluorescence (IF) detection. The nucleus was stained with 4′,6-diamidino-2-phenylindole (DAPI, blue), and *LGR5* was stained with anti-*LGR5* (green). *Scale bars*: 100 μm

Intestinal tissues were collected from chicken embryos of different ages (E9, E11, E13, E15, and E18). E9 chicken embryos developed rapidly, and the growth rate of the intestinal tissue increased with age (Fig. 1A). At E13, E15, and E18, the intestinal segments were significantly longer and had grown rapidly, indicating a completely differentiated intestine. As the embryo ages, the intestine gradually becomes longer and larger with more prominent structures present. This reflected the ability of early embryonic intestinal cells to proliferate and differentiate. H&E-stained sections were used to analyze the development of intestinal cells at early embryonic stages (Fig. 1B). The results showed that the intestinal lumen was present at E9 and intestinal microvilli were formed. Although their number was small, the depth was shallow and the length was relatively long. At E11, the number and depth of small intestinal crypts and villi increased. At E13, the number of intestinal crypts and villi increased significantly and were arranged in an orderly manner. At E15, the lumen was further enlarged and the crypts between the intestinal villi were deepened. At E18, the intestinal lumen was larger and wider and the intestinal crypts were closely connected. Furthermore, the number and length of the intestinal villi significantly increased.

Avian embryonic epithelial cells are differentiated from the most primitive intestinal stem cells and express the stem cell marker *LGR5* [29]. The intestinal tissues of E9, E11, and E15 embryos were selected for the localization and enumeration analysis of *LGR5*-expressing stem cells. *LGR5*-positive cells in the E9 intestinal tissue were distributed evenly on the surface of the intestinal villi and crypts, with a low number but high purity. The number of intestinal cell crypts and stem cells in E11 and E15 embryos gradually increased (Fig. 1C).

### Establishment of immortalized AIECs using *SV40T* transfection technology

**Fig. 2:**
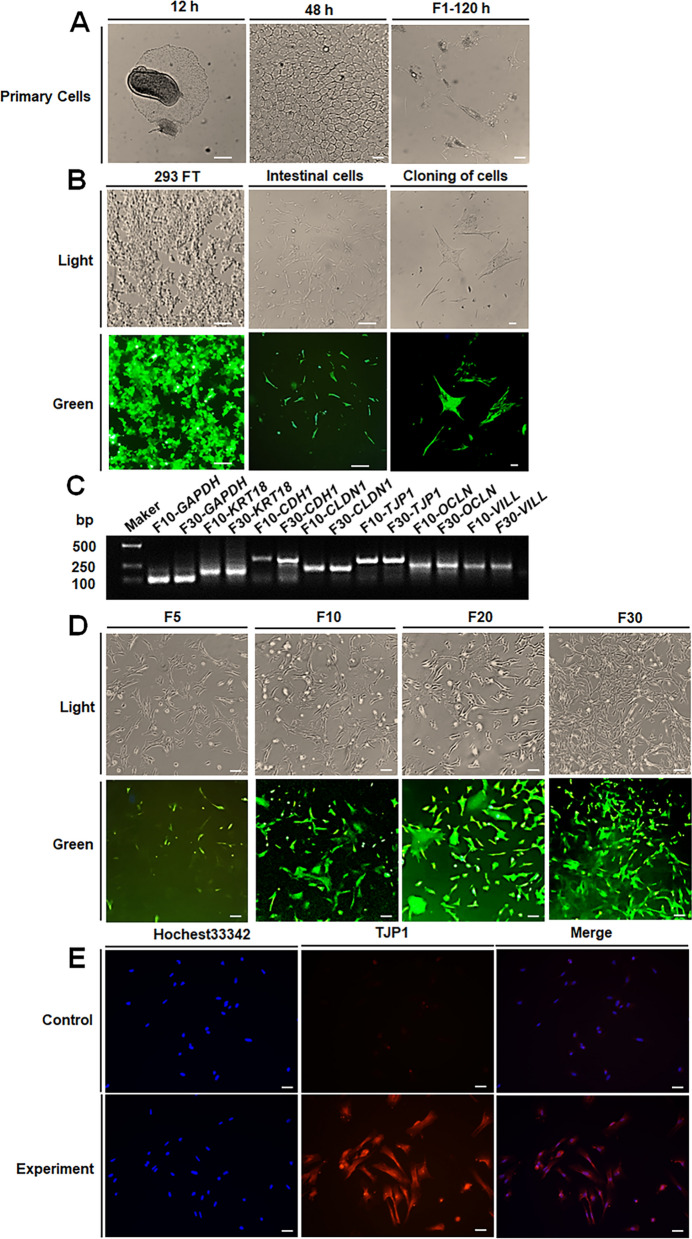
Morphology of primary epithelial cells isolated from E9. **A** Cellular morphology in 12, 48, and 120 h of culture after isolation. **B** *SV40T* antigen was successfully transfected into embryonic kidney epithelial cells (293FT) and integrated into the small avian intestinal epithelial cell line (AIEC) of E9 embryos.** C** F10 and F30 AIEC marker genes (*KRT-18*, *CDH1*, *CLDN1*, *TJP1*, *OCLN*, and *VILL*) expression detection. **D** Morphology of F5, F10, F20, and F30 AIEC (light/green). **E** Representative images of immunofluorescence staining of F30 AIEC for TJP1 showing a single cell layer, including nuclei of cells stained in blue (HoChest33342) and TJP1 in red. The images were captured with a fluorescence microscope. *Scale bars*: 50 μm

Primary small intestinal epithelial cells from avian embryos were isolated using the tissue block culture method, and cell morphology was observed under an inverted microscope (Fig. 2A). Within 12 h of the initial culture, single-cell clusters began to adhere to the culture flask. After 48 h, the cells proliferated rapidly and two different morphological types were observed: one type was epithelioid and polygonal, and the other type was fibrous. After the first passage and 120 h of subculturing, the cells were senescent, elongated, and pointed in shape, with many vacuoles.

To establish the AIEC, the pcDNA-Large T-IRES-CoGFP plasmid was constructed and co-transfected into 293FT cells. After 2 days of infection, cells from different embryonic stages completed the viral infection, but only E9 could adapt to long-term culture (Fig. 2B). Single-clone cells were isolated by gradient dilution and collected in 96-well plates. After 2–3 weeks of culture, the cell fusion rate was as high as 80% and most cells were positive for GFP. Preliminary assessment of cell morphology showed similarities to epithelial cell clones (Fig. 2B). To determine whether the cell line was stable, continuous passage, fluorescence observation and PCR detection technology were used to detect the expression of epithelial cell marker genes at different time points. F10 and F30 passage cells expressed *KRT-18*, *CDH1*, *CLDN1*, *TJP1*, *OCLN*, and *VILL* marker genes (Fig. 2C). Through passage culture, we found that the AIEC of F5, F10, F20, and F30 had a uniform distribution, strong vitality, and complete morphology and structure (Fig. 2D). Immunofluorescence results showed that the marker protein TJP1 was positively expressed, whereas the negative control group showed no signal. AIEC stably expressed the intestinal epithelial marker TJP1 (Fig. 2E).

### Biological analysis of different passages of AIECs showed that most differentially expressed genes were associated with cell cycle regulation

**Fig. 3:**
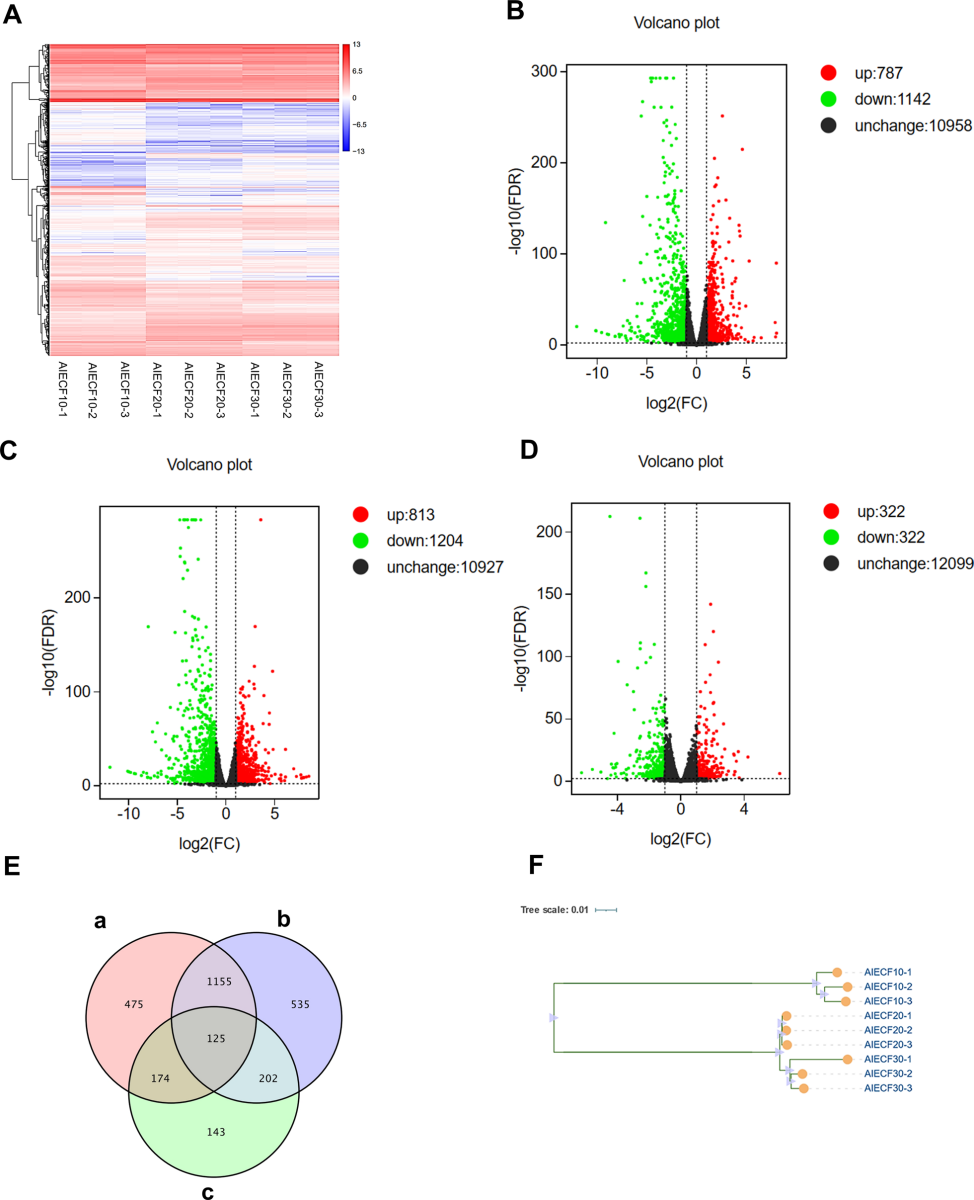
Comparison of gene expression. **A** Heatmap of differentially expressed genes (DEGs). **B** F10 and F20 volcano plots of DEGs. **C** F10 and F30 volcano plots of DEGs. **D** F20 and F30 volcano plots of DEGs. **E** Venn diagram showing co-expression of differential genes (a, AIECs F10-F20; b, AIECs F10-F30; c, AIECs F20-F30). **F** Phylogenetic tree of F10, F20 and F30; log2(FC) < 1, adjusted *P*-value < 0.05)

The transcriptome was sequenced and analyzed from the mRNA of different passages of AIEC cultures. Cluster analysis revealed that different passages of cells had different transcriptional profiles. Notably, the relative gene expression differences between F20 and F30 were lower than those between F10 and F30, and the expression was more stable (Fig. 3A). At F10 and F20 passages, the total number of DEGs was 1929. A total of 787 genes showed upregulated expression and 1142 genes showed downregulated expression (Fig. 3B). Comparisons between F10 and F30 revealed significant DEGs. A total of 813 genes were upregulated, and 1204 genes were downregulated (Fig. 3C). There were 644 significant DEGs between the F20 and F30 groups, with 322 genes showing upregulation and 322 genes showing downregulation (Fig. 3D). A Venn diagram revealed that 125 DEGs between F10, F20, and F30 of cultured AIECs were expressed in all three passages (Fig. 3E). Phylogenetic tree analysis indicated that F10, F20, and F30 cells were divided into two clades. F10 cells were separated into a single branch, whereas F20 and F30 cells were combined into the same branch, indicating that the cell relationship between F20 and F30 was relatively close (Fig. 3F). As the mRNA expression profiles of F10 and F30 were more diverse, they were chosen for further investigation of the functions of DEGs.

### GO and KEGG enrichment analysis revealed alterations in cell cycle-related genes expressed by AIECs in in vitro culture conditions

**Fig. 4:**
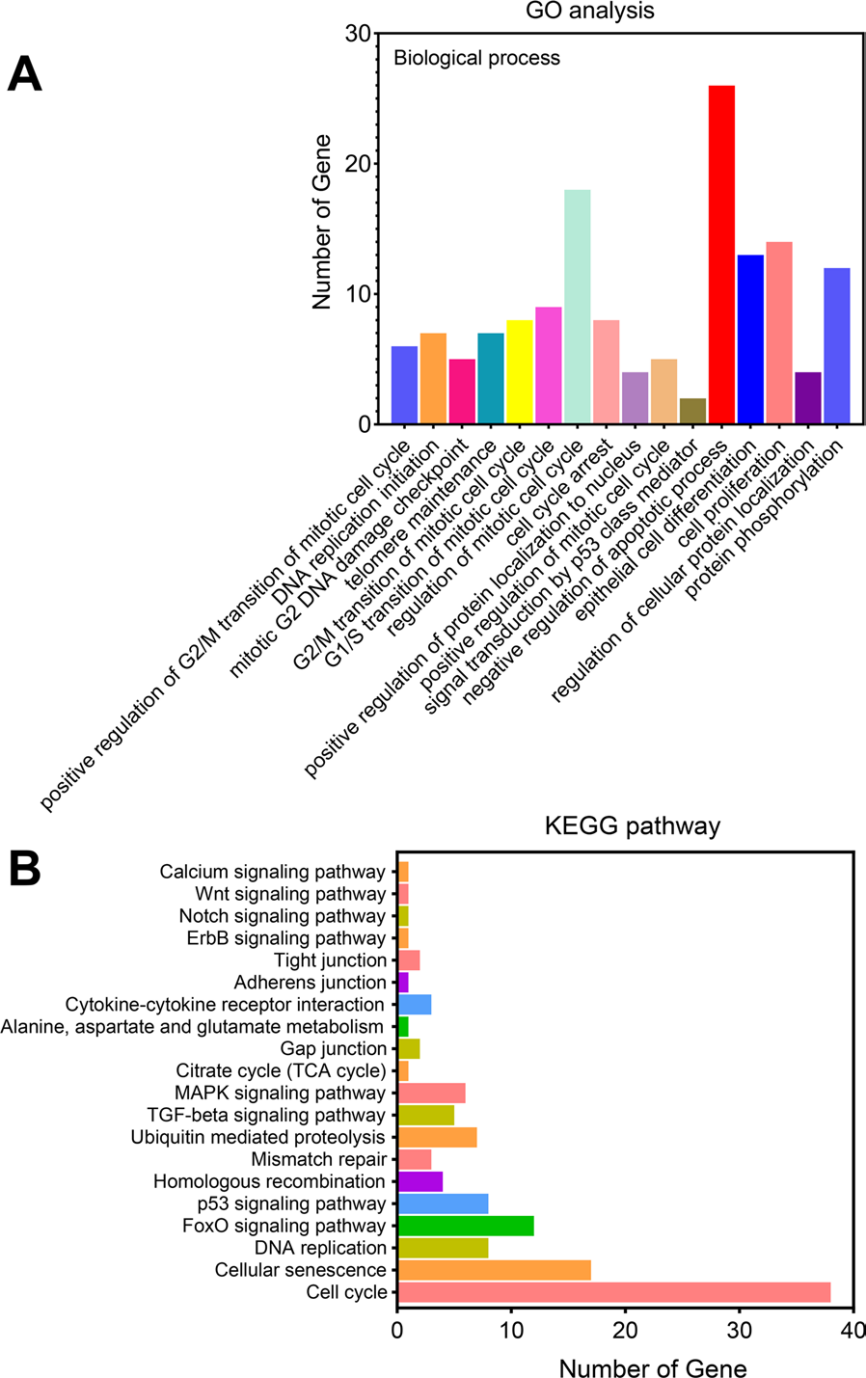
Enrichment analysis of avian intestinal epithelial cell line (AIEC) in F10 and F30. **A** Gene Ontology (GO) analysis. **B** Kyoto Encyclopedia of Genes and Genomes (KEGG) enrichment analysis of AIECs

GO functional annotation enrichment analysis revealed significant changes in 1496 gene sets out of a total of 16,132 gene sets based on the DEGs compared between the F10 and F30 passages. To further verify the reliability of our findings, we analyzed the top 20 significantly enriched categories in biological functions, biological processes, and cellular components (Fig. 4A, B). DEGs were broadly enriched for GO terms associated with negative regulation of biological processes and cell components mainly participated in the regulation of cell cycles. Through the GO analysis of F10 and F30, we found that the possible biological processes that DEGs were mainly involved in were positive regulation of G2/M transition of the mitotic cell cycle, DNA replication initiation, mitotic G2 DNA damage checkpoint, telomere maintenance, G2/M transition of mitotic cell cycle, G1/S transition of mitotic cell cycle, regulation of mitotic cell cycle, cell cycle arrest, positive regulation of protein localization to nucleus, positive regulation of the mitotic cell cycle, signal transduction by p53 class mediator, negative regulation of the apoptotic process, epithelial cell differentiation, cell proliferation, and regulation of cellular protein localization and protein phosphorylation. Next, KEGG enrichment analysis was conducted on F10 and F30, and it was found that the differential genes were mainly enriched in the cell cycle. Successful establishment of AIECs mainly involves regulating the cell cycle. The upregulation of genes that express cell cycle signaling pathways and other related pathways plays a key role in the downregulation of genes related to cell aging and p53 signaling pathways. This provided a basis for establishing the cell lines.

**Fig. 5:**
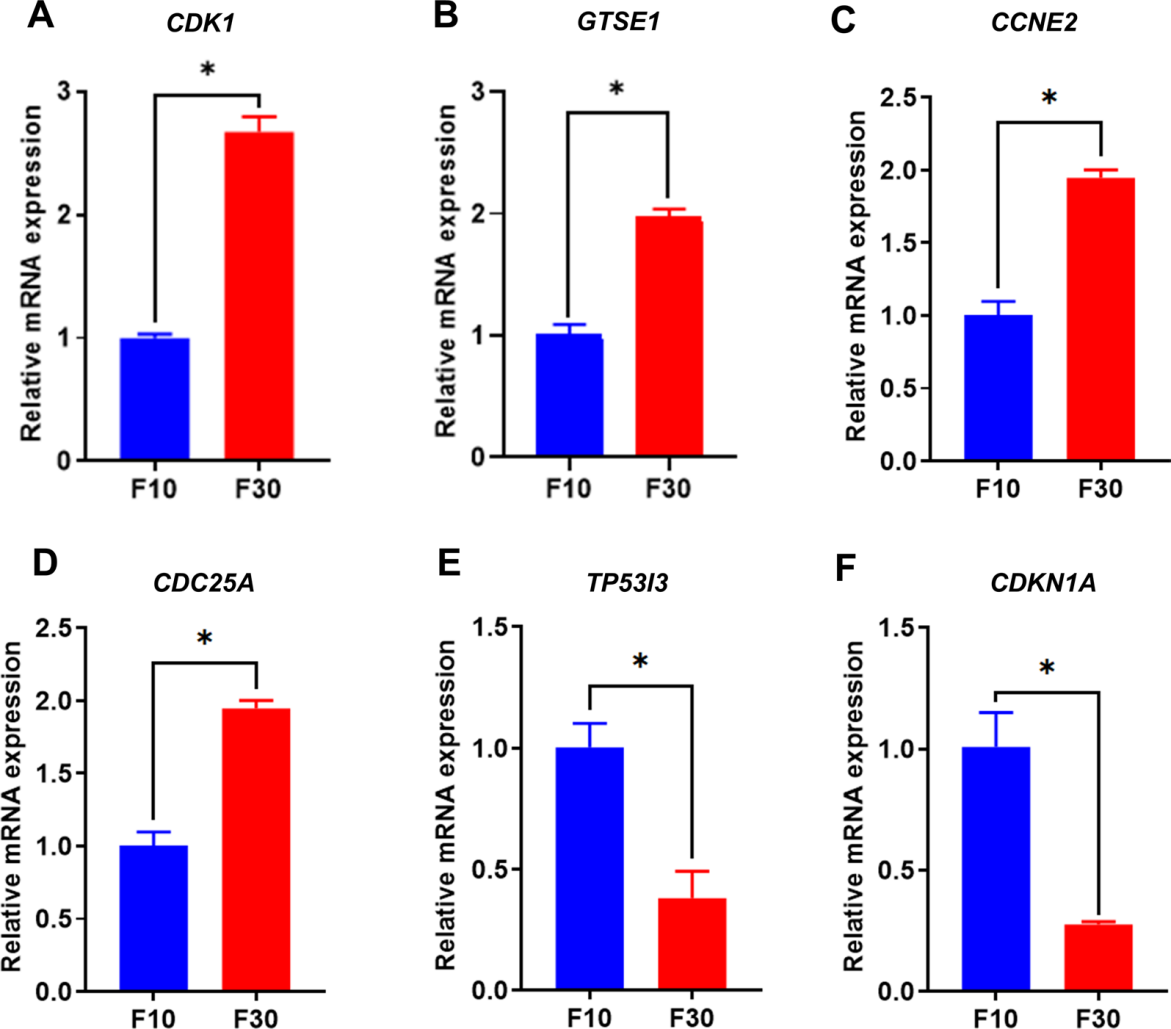
The expression of cell cycle genes in the F30 of the avian intestinal epithelial cell line (AIEC) was detected and measured by quantitative polymerase chain reaction (qPCR). **A–D** Upregulated gene expression in F30 AIECs. **E, F** Downregulated gene expression in F30 AIECs

To validate the DEGs and pathways revealed by RNA-Seq in different passages of AIECs, the expression levels of target genes involved in the cell cycle were examined by qPCR. Between F10 and F30, we observed upregulation of *CDK1*, *GTSE1*, *CCNE2*, and *CDC25A*, which promote cell proliferation. Conversely, *CDKN1A* and *TP53I3*, which inhibit cell apoptosis, were downregulated in F30 AIECs, suggesting that F30 had already exhibited a stable cell passage condition (Fig. 5A–F). In summary, our findings demonstrate consistent alteration in the majority of cell cycle-related genes and the biological processes of AIECs, suggesting their potential adaptability for future in vitro culture studies.

### Efficient functional response to *E. tenella* infection, with higher passage of AIECs being more conducive to the early invasion and development of* E. tenella*

**Fig. 6:**
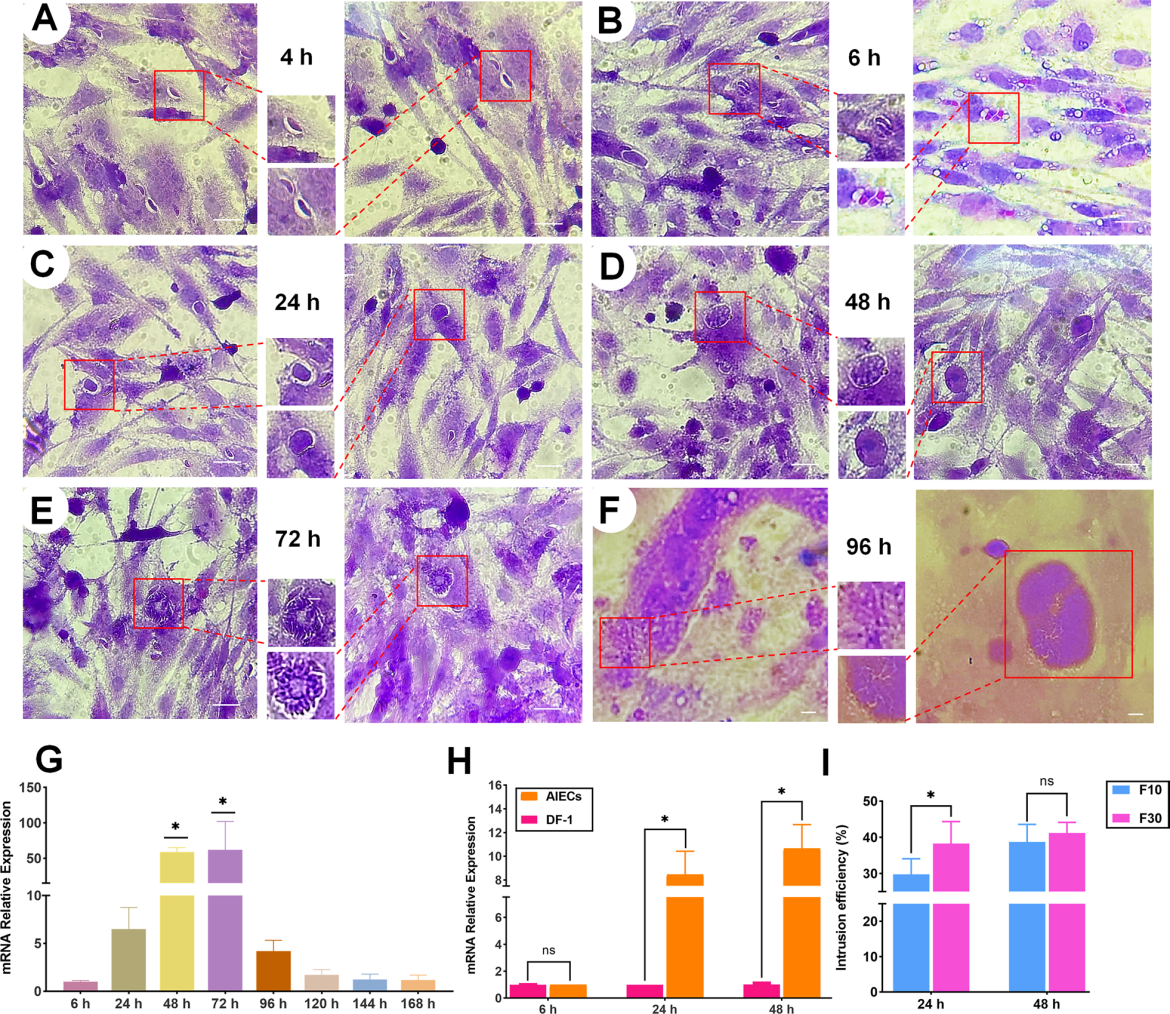
Infection and development of *E. tenella* in AIECs. Cells were infected with *E. tenella* sporozoites. **A** After 4 h of infection, most sporozoites could find and invade AIECs. **B** After 6 h of infection, the invasion was successful and the sporophyte formed. **C **After 24 h of infection, the formation of mononuclear trophozoites were observed. **D** After 48 h of infection, the formation of first-generation immature schizonts was observed. **E** After 72 h of infection, the formation of first-generation mature schizonts with budding merozoites was observed. **F** After 96 h of infection, the second-generation schizonts and merozoites were observed (H&E staining). **G** At different time points (6, 24, 48, 72, 96, 120, 144, and 168 h), the effect of *E. tenella* activity on the endogenous developmental stage was evaluated by measuring gene expression of *EtGAPDH*. **H** Infection efficiency of* E. tenella* sporozoites inoculated with DF-1 and AIECs at 6 h, 24 h and 48 h. **I** Inoculation of *E. tenella* sporozoites on F10 and F30 AIECs at 24 h and 48 h. Scale bars: 20 μm

*Eimeria tenella*, an intestinal pathogen, was used to test the susceptibility of AIECs to infection. The influence of *E. tenella* invasion on epithelial cells was first assayed by exposing an AIEC monolayer to sporozoites for 4, 6, 24, 48, 72 , and 96 h, studying endogenous development after H&E staining. Most *E. tenella* sporozoites with good vitality could quickly find AIECs and invade them within approximately 4 h post-infection (p.i.) (Fig. 6A). After 6 h, the sporozoites colonized an area near the nucleus and completed their invasion (Fig. 6B). After a parasitophorous vacuole was formed, the sporozoites developed into oval-shaped trophozoites and started their intracellular periods. The trophozoites began to enlarge, became round, and were transformed into initial infective form with refractive properties 24 h p.i. (Fig. 6C). After 48 h, first-generation immature schizonts with refractive index, a characteristic of mononuclear trophozoites, were observed (Fig. 6D). At approximately 72 h p.i., chrysanthemum-shaped mature schizonts containing merozoites were observed (Fig. 6E). After 96 h, the second-generation schizonts were observed, which were slightly larger than the first-generation schizonts and presented as an irregular circle (Fig. 6F). However, we were unable to observe the obvious oocysts in the late stage, although we could not rule out the possibility that they were present at levels below our ability to detect them.

In addition, the effect of *E. tenella* activity on the endogenous developmental stage was evaluated by measuring gene expression of *E. tenella GAPDH*, at different infection time points. From 6 h of infection, the expression level of *EtGAPDH* was gradually increased at 24 h p.i., 48 h p.i., and with a peak at 72 h p.i. It began to decrease significantly from 96 h and became stable after 120 h (Fig. 6G). These results suggest the possibility for supporting parasite asexual stage development with AIECs.

Furthermore, the infection burden efficiency of *E. tenella* on AIECs was compared with that in DF-1, another cell line of natural host origin. The infection burden efficiency at 6, 24, and 48 h was much higher than that in DF-1, and the infection efficiency at 24 and 48 h was significantly higher (Fig. 6H). However, direct inoculation of *E. tenella* sporozoites into the AIECs of F10 and F30 resulted in a significant difference in the infection burden (Fig. 6I). AIECs at F30 were more conducive for the early invasion and development of *E. tenella* (24 h). After 48 h of infection, F30 was superior to F10; however, the difference was not statistically significant. These results indicate that AIECs stably changed the cell cycle and energy metabolism regulation, which shows greater advantages in the infection and invasion efficiency of *E. tenella* early development.

## Discussion

The intestine is the main organ for digestion and absorption, participates in the regulation of nutrient intake, and provides a barrier against macromolecules and pathogenic microorganisms. Owing to the complexity of intestinal physiological mechanisms and the lack of an appropriate in vitro model, studies on pathogen–host interactions are limited [1, 30]. In this study, the intestinal tissues and organs of avian embryos of different embryonic ages were separated. Embryos and intestinal tissues developed rapidly during the incubation process, and cell proliferative ability increased with increasing incubation time. The epithelium of the small intestine is composed of a monolayer of columnar cells, many of which have well-developed microvilli with brush-like edges, and hundreds of dry cells present in crypts [31]. During development, cells undergo extensive proliferation, and many cell types appear, forming an intestinal structure. The epithelial cells at the top of the villus are highly differentiated, have a short lifespan, and are prone to aging. Under these circumstances, *LGR5* stem cells at the bottom of the crypts constantly supplement the epithelial cells and maintain intestinal integrity [29]. To observe the development of the intestinal tissue more clearly, tissues at E9, E11, and E15 were selected for morphological observations. H&E staining showed that the intestinal crypts of chicken embryos were not formed at E9, but microvilli were formed at this stage. The internal structure formed at E11 and changed significantly at E15, primarily because the number of crypts increased. Immunofluorescence localization analysis showed that *LGR5* stem cells at E9 were present, located on the surface of intestinal villi and crypts in a small number, but with high purity. The number of intestinal tissue cells at E11 and E15 increased along with the number of crypts and stem cells. Intestinal cells at the E9 stage did not differentiate during early embryonic development, and the most primitive stem cells played the main effect. This suggested this would be a good stage for establishing a cell line. The proliferative ability of cells in embryonic stages is higher than that in adult tissues, and the probability of successful cell line establishment is much greater [2, 32, 33].

Compared to other cell models, primary cells are closer to the original state of the organism. However, they are more difficult to obtain and maintain, and their lifespan is limited. Epithelial cells possess the fastest proliferation and renewal abilities [34]. The average lifespan of chicken epithelial cells is 3–5 days, and primary epithelial cells do not exceed this period in vitro [35, 36]. Senescence, apoptosis, and vacuoles in chicken embryonic epithelial cells were observed 120 h after in vitro culture, restricting the establishment of cell lines. *SV40T*-induced immortalization can inhibit the cell cycle by inactivating *p53* and *pRB*, thereby overcoming this challenge [37]. We hypothesized that AIECs could be obtained using transgenic large T technology to prolong their lifetime in vitro. Our results showed that large T-EGFP was successfully expressed, and AIEC was successfully cultured in vitro continuously for more than 6 months, with more than 30 passages. qPCR analysis revealed that epithelial markers (*KRT-18*, *CDH1*, *CLDN1*, *TJP1*, *OCLN* and *VILL*) were stably expressed in F10 and F30 cells, which is consistent with previous research results [38]. Many microorganisms exist in the intestinal crypts to ensure effective nutrient absorption and treatment of the intestine. Intestinal epithelial cells can generate various types of barriers to protect the intestine from invading pathogenic microorganisms and thus are an important component of gastrointestinal mucosal immunity [39]. Tight junctions (TJs) mainly act as dynamic permeability barriers, preventing potentially harmful substances or pathogens from entering and absorbing nutrients, ions, and water [40, 41]. TJ proteins control resistance to bacterial toxins and pathogens [42]. The TJ protein TJP1 is a typical membrane-associated guanylate kinase (MAGUK) family protein that mainly acts as a scaffold at specific locations within cells [43]. In our study, F30 AIECs were selected for immunofluorescence detection, and the marker protein TJP1 was positively expressed, whereas the negative control group showed no signal. AIECs are produced from undifferentiated cells in the small intestinal villi and express TJ proteins that are not expressed by undifferentiated basal cells, thus forming a physical barrier in the intestinal epithelium [38]. Previous studies have reported that TJP1 is mainly responsible for the protein network between actin and tight junction proteins (such as occludin and claudin), maintaining cell integrity, and participating in paracellular closure and membrane domain differentiation [44]. Early studies found that after 4 days of incubation, the TJ protein occludin showed weak expression in the intestine and then gradually increased. At the 11th day post-hatching, it was only expressed at the top of the epithelial cells [45]. Studies have also reported expression of claudin-1 and claudin-3 in the intestinal epithelium after 5–8 days of embryonic development [46, 47].

To explore the physiological effects of continuous passaging on AIECs, F10, F20, and F30 cells were selected for bulk RNA sequencing. Many significant DEGs are involved in the regulation and signal transduction of key genes and pathways, particularly in the promotion of cell proliferation and differentiation [37]. Enrichment analysis of DEGs functions illustrated that these genes have potential regulatory effects on cell cycle operation and functional metabolism. The *SV40T* gene directly promoted the upregulated expression of cell cycle genes (*CDK1*, *GTSE1*, *CCNE2*, and *CDC25A*) during the establishment of cell lines, allowing AIECs to cross the crisis period and maintain stable proliferation in vitro. Simultaneously, some apoptotic genes were downregulated (*CDKN1a* and *TP53I3*), which maintained the stability of the cells under normal metabolic conditions. In some cell types, excessive activation of cell cycle processes may lead to cell death [48]. The normal cell cycle is an important requirement for homeostasis. The cyclin-dependent kinase *CDK1*, a serine/threonine kinase that participates in the transition of the G1/S and G2/M phases, regulates cell proliferation, differentiation, aging, apoptosis, and other physiological states. *CDK1* is highly expressed in tumor cells, where it promotes cell proliferation by regulating the G2/M phase to induce epithelial-to-mesenchymal transformation [49]. G2 and S phase expressed-1 (*GTSE1*) is encoded by a gene located on chromosome 22q13.2–q13.3, which is specifically expressed only in the S and G2 phases. It inhibits *p53*-induced apoptosis by promoting *p53* degradation [50]. Related studies have shown that *GTSE1* can negatively regulate *p53* by stimulating *p53* to relocate to the cytoplasm and inhibiting *p53*-induced apoptosis when DNA damage occurs [51]. Cyclin E2 (*CCNE2*) is the second member of the E-type cyclin-dependent kinase (CDK) family and participates in the G1/S phase transition and cell proliferation [52]. In prostate cancer, *CCNE2* is upregulated and considered a tumor-promoting protein [53]. This study found that *CCNE2* was upregulated in AIECs of the F30 and promoted cell proliferation under normal operation of the cell cycle, consistent with other reports [54].

Cyclic phosphatase *CDC25A* is a key regulator of cell cycle progression and is considered a carcinogenic gene. Its function involves regulating the phosphorylation of CDK complexes [55]. It activates the CDK cyclin complex, which regulates cell cycle transition during normal cell division and ensures genetic stability in case of DNA damage [56]. Our study found that upregulation of *CDC25A* in AIECs not only regulated the early G1/S transition but also regulated the late G2/M transition and maintained cell proliferation. The cell cycle inhibitor *CDKN1A* was originally identified as a tumor suppressor and CDK inhibitor [57, 58]. It was later found to be involved in cell death, DNA replication/repair, gene transcription, cell movement, and other important processes [59]. In this study, *CDKN1A* was downregulated in F30 AIECs, maintaining the normal operation of the cell cycle and promoting cell proliferation. Relevant studies have found that in mouse embryonic fibroblasts (MEF), the G1/S phase block is impaired after DNA damage if *CDKN1A* is missing [60, 61], and *CDKN1A* plays a role in maintaining the G2 phase block [62, 63]. The main role of *CDKN1A* at the G1 checkpoint is to inhibit the activity of cyclin E and cyclin A/CDK2 in the G1/S phase and promote G1/G2 blockade by mediating the degradation of cyclin B in response to DNA damage [64, 65]. *CDKN1A* is an important regulator of cell cycle checkpoints that ensures normal cell division [58, 66]. *TP53I3* is a unique quinone oxidoreductase that participates in DNA damage response and *p53*-mediated apoptosis [67–69]. In ovarian cancer, *TP53I3* is transcriptionally activated by *p53* and is thought to play a role in the DNA damage response and apoptosis induced by reactive oxygen species [70]. The P53 protein translated by the *TP53I3* gene is an important regulator of cell growth and proliferation and DNA damage repair. When DNA is damaged, P53 locks cells in the G1/S phase, which is then able to repair the damage. If it cannot be repaired, cell apoptosis is initiated. We found that *TP53I3* was downregulated in AIECs, thereby inhibiting apoptosis and promoting proliferation. Relevant studies have shown that the downregulation of *TP53I3* is involved in the *p53*-dependent cell death signaling pathway [71], which also suggests that the overexpression of *TP53I3* may be related to apoptosis and involves the *p53* network [72].

The life cycle of *Eimeria* is complex, and includes multiple asexual and sexual stages. Sporozoites rapidly develop into schizonts after entering the epithelial cells. A large number of merozoites are released when a schizont matures and ruptures, leading to the rapid necrosis, disintegration, and shedding of mucosal epithelial cells [73]. A stable in vitro cell culture model is crucial for studying the mechanism underlying coccidian invasion and host interactions. There are limitations to in vitro models, which can only simulate some stages of the complex life cycle [74], and most rely on the development of primary cells. It was found that due to inappropriate cell culture models, the sporozoite formation rate was lower than that obtained in vivo [8, 13]. Purified sporozoites can be inoculated into the primary kidney cells and cecal epithelial cells of birds to develop into oocysts. However, primary cells cannot be passaged, each experiment is cumbersome, and the results vary. These drawbacks limit research on coccidian pathogenesis. Compared with the primary cells, cell line models have a clear background and are preferred by many researchers. For example, MDBK is the choice for drug screening of *E. tenella* [22]. However, MDBK cells are not derived from natural hosts, which limits research on the mechanisms of coccidiosis.

In this study, AIEC models were established using E9 avian embryos. Invasion was successfully completed 4–6 h after inoculation with sporozoites. After 24 h, the sporozoites developed into oval monokaryon trophozoites. After 48 h, the first immature schizonts with refractive properties were observed. After 72 h, protoplast globules split from the middle, and mature schizonts appeared, which is consistent with prior research [9]. When sporozoites were infected with AIEC and DF-1 cells simultaneously and cultured for 6, 24, and 48 h, the infection efficiency of AIEC was significantly better than that of DF-1, and the effect was most significant at 48 h. Subsequently, we found that there were certain differences in AIEC inoculated at F10 and F30 at different time points, and F30 cells were more conducive to coccidian invasion and development. This may be because the invasion of *E. tenella* is associated with the expression of cell cycle genes, as *E. tenella* regulates the cell cycle progression of the host cell during invasion, thereby allowing the host cell to enter a state conducive to the survival of *E. tenella*. Specifically, *E. tenella* can promote its invasion and survival by activating or inhibiting the expression of cell cycle regulatory factors, altering cell cycle progression, and controlling cell apoptosis [75, 76]. In addition, the cell cycle-related pathways in GO and KEGG were confirmed to be beneficial for the invasion of *E. tenella* and promoted the transport and metabolism of intracellular substances, thereby enhancing the ability of *E. tenella* to invade cells.

## Conclusions

In the present study, we successfully established a stable AIEC. We induced cells to avoid the danger period for apoptosis by transducing the *SV40T* gene to regulate the expression of cyclin, maintain stable proliferation of cells, increase the number of passages, and complete long-term culture in vitro. This cell line retained the morphological and functional characteristics of primary chicken embryo epithelial cells and was able to sustain *E. tenella* infection. Overall, the AIECs established in this study will provide favorable research materials for investigating cell invasion, early development, and mechanisms of interaction with the host parasite, and will facilitate future research on vaccines and treatments.

## Data Availability

All RNA-Seq data were deposited in the GEO database (accession number: PRJNA995178; https://www.ncbi.nlm.nih.gov/geo). All data generated or analyzed during the study period are included in this article.

## Abbreviations

AIEC: Avian intestinal epithelial cell line
DF-1: Chicken fibroblast cell line
DEGs: Differentially expressed genes
MDBK: Bovine kidney cells
LMH: Chicken liver cancer epithelial cells
CLEC-213: Chicken lung epithelial cell line
293FT: Embryonic kidney epithelial cells
DMEM: Dulbecco’s modified Eagle medium
